# Challenges, opportunities, and potential roles of the private primary care providers in tuberculosis and diabetes mellitus collaborative care and control: a qualitative study

**DOI:** 10.1186/s12913-022-07612-3

**Published:** 2022-02-17

**Authors:** Merita Arini, Dianita Sugiyo, Iman Permana

**Affiliations:** 1 Department of Family Medicine and Public Health; and Master of Hospital Administration, Universitas Muhammadiyah Yogyakarta, Jalan Brawijaya, Tamantirto, Kasihan, Bantul, 55183 Indonesia; 2 Department of Public Health Nursing, School of Nursing, Faculty of Medicine and Health Sciences; and Muhammadiyah Steps, Universitas Muhammadiyah Yogyakarta, Jalan Brawijaya, Tamantirto, Kasihan, Bantul, 55183 Indonesia; 3 Master of Nursing; and Center of Biotechnology and Halal Studies, Universitas Muhammadiyah Yogyakarta, Jalan Brawijaya, Tamantirto, Kasihan, Bantul, 55183 Indonesia

**Keywords:** Chronic disease, Public-private mix, Private primary care, Tuberculosis, Diabetes mellitus

## Abstract

**Introduction:**

The comorbidity of tuberculosis and diabetes mellitus (TB-DM) is a looming global co-epidemic problem. Despite the Indonesian Government’s ongoing effort to impose regulation for collaborative TB-DM management, the involvement of private primary care providers (PPCs) has not been considered before the COVID-19 pandemic. This study aimed to capture the PPCs’ existing practices and explore their challenges, opportunities, and potential roles in the collaborative TB-DM services and control.

**Methods:**

A descriptive qualitative research design was used to collect data. Two Focus Group Discussions (FGDs) were conducted with 13 healthcare workers (HCWs) from different private clinics and eight private/solo general practitioners (GPs) from Yogyakarta City, Indonesia. We triangulated these data with data from FGDs of HCWs community health centers (CHCs) and in-depth interviews of three regional health regulators, five hospitals staff members, and a representative of national health insurance. The discussions were audio-recorded, transcribed verbatim, and subjected to thematic analysis.

**Results:**

PPCs have not been initiated into the implementation of the collaborative TB-DM programme. The themes identified in this study were health system-related barriers, knowledge and perception of HCWs, lack of implementation of bi-directional screening, and needs of multisector role. The potential roles identified for PPCs include involvement in health promotion, bi-directional screening, patient referral, and data reporting according to the TB-DM programme indicators. However, more thorough improvement of PPCs’ capacity and logistic supplies are needed to provide comprehensive TB treatment.

**Conclusion:**

Although PPCs’ involvement in implementing collaborative TB-DM services has yet to be considered, their potential role should not be neglected. Therefore, it is essential to increase their involvement by enhancing their capacity and improving the Public-Private Mix. PPCs’ engagement should be initiated and maintained to ensure the sustainability of the programme.

## Introduction

In recent years, tuberculosis (TB) and diabetes mellitus (DM) have become two of the ten leading causes of death in the world [1]. The increasing comorbidity of the two diseases is a looming global co-epidemic [2]. It represents a complicated and growing problem, which combines a communicable and a non-communicable disease in a problematic context. DM is a significant TB risk factor; its prevalence among TB patients is between 1.9 and 45% and continues to increase [3, 4]. Meanwhile, TB prevalence among DM patients ranges from 0.38 to 14% [4]. Hence, the World Health Organization (WHO) declared in 2011 that the collaborative programme for TB-DM care and control is one of its TB eradication strategies, and included it as an essential part of the efforts to achieve Sustainable Development Goals (SDGs) [5, 6].

Indonesia still has the second-highest number of TB cases in the world [3]. Despite the increase in reported cases of TB, there was still a significant difference between the reported number of incident cases (7.0 million) and the estimated number of new cases (10 million) in 2018. This discrepancy is attributable to both underreporting, low diagnosis, and case identification. Indonesia is responsible for 10% of this gap in the world [3]. This situation is exacerbated by TB being closely correlated with DM in Indonesia [7]. As well as being known for worsening TB outcomes and treatment failure, DM often leads to TB patients being unrecognized due to non-typical symptoms [8]. This difficulty hampers the identification of cases since it often causes delays in diagnosis in Indonesia [9]. The rising incidence of DM adds to Indonesia’s double burden of diseases. Indonesia is ranked 7th highest among adults with DM in the world, and its prevalence is estimated to continue to grow year on year [10]. The incidence of TB-DM may become higher if preventive measures and control efforts are not implemented.

Challenges emerged in starting collaborative TB–DM care and control in Indonesia from its different characteristics from other collaborative services in this country. The collaborative TB–DM service integrates community health-based TB management activities, and DM services initially focused on individual health. Meanwhile, several health services have been integrated, generally collaborative community health-based programmes, such as TB–HIV, sexually transmitted diseases programme with MCH (maternal–child health) services, and Posyandu (an integrated service post) for child health motoring and immunisation.

TB services in Indonesia are managed programmatically, with public primary health care (Puskesmas/Community Health Centres [CHCs]) as the programme’s backbone. CHCs are obliged to carry out case tracking, treat new and relapsed pulmonary TB cases without complications, carry out health promotion activities, and conduct surveillance. Meanwhile, public and private hospitals that collaborate with the national TB programme (NTP) act as referral health facilities that handle TB cases with complications that require specialist management. All TB health service providers must record and report their activities to the NTP. Private primary care providers (PPCs) have not significantly been involved in TB management in Indonesia.

In Indonesia, DM management was previously a curative health service for individuals in primary care and hospitals. DM began to be managed programmatically in CHCs in the last decade. Since it is a new strategy, the DM programme still necessitates a number of processes to ensure that change management is implemented appropriately. DM is also one of the non-communicable diseases prioritised for comprehensive management in primary care and hospitals since PROLANIS (the chronic disease care programme) was launched by BPJS-Kesehatan (Indonesia’s national health insurance agency) [11].

BPJS-Kesehatan is the major payor for health services in Indonesia, although healthcare for the two diseases has different financing mechanisms. TB care is generally financed by the NTP with various funding sources from international institutions and governments [12]. BPJS-Kesehatan has a stake in financing TB services in hospitals. Generally, healthcare financing for DM is managed by BPJS-Kesehatan, both in primary and referral services. However, various types of funding for DM care are currently starting to play a role.

Indonesia’s current health care structure is supported by a vast private sector [13]. Most Indonesian TB patients initially seek care from PPCs; however, these health providers have limited diagnostic tools and TB treatment supplies, and health workers’ (HCWs’) capacity and compliance are inadequate [14–16]. Moreover, PPCs tend to lag in notification of case and the quality of TB care [14–17]. On the other hand, PPCs have enrolled some DM patients in PROLANIS, while other DM patients are managed conventionally [13]. Although the government has issued guidelines and policies since 2015 for collaborative TB–DM management, it has not been systematically carried out in public and private health services.

Given the large number of patients seeking treatment at PPC and their services, specific approaches are needed to increase mutual detection, prevention, and management between TB and DM. The government of Indonesia initially established regulations and programmes to boost the private sector’s role in TB management through a Public-Private Mix (PPM) [14, 18]. The PPM was crucial since extensive evidence indicates that failure to involve private providers used by suspects and diagnosed TB patients hinders case detection, causing delays in diagnosis, contributing to inadequate and ineffective treatment, increasing drug resistance, and imposing unnecessary financial burden on patients [19]. However, these efforts have not yet begun to be applied to collaborative TB-DM management. Therefore, this study’s objectives were to assess existing practices, explore challenges, opportunities, and potential roles that PPC can play in collaborative TB-DM control and care.

## Methods

### Study setting

This study was conducted between June and August 2019 in Yogyakarta City, an urban area with the highest population density district in the Province of Yogyakarta Special Region, Indonesia. With an area covering 35.2 km^2^, the population comprises 412,726 inhabitants in 2018 [20]. The medical services of Yogyakarta city are provided through a network of 18 Puskesmas/ community health centers (CHCs), 20 hospitals, 127 clinics, and hundreds of registered general practitioners (GPs) who work in private practices [20].

### Participants

Purposive sampling using the criterion sampling technique was applied to select participants. Two focus group discussions (FGDs) were conducted involving 13 healthcare workers (HCWs) from different private clinics and eight private/ solo GPs. The representative health practitioners from private clinics and solo GPs were invited from the list of health facilities registered with the Yogyakarta City Health Office. They were selected using the inclusion criteria: providing non-specialist health services; serving a dense patient population; and/or cooperating with national health insurance (BPJS-Kesehatan).

### Study design and data collection

This study was a descriptive qualitative study focused on private providers’ perspectives and experiences in TB-DM care and control. As a part of umbrella research on disease management in Yogyakarta City, this study’s primary unit of analysis was private providers in two separate FGDs: solo GPs and private clinics. We also triangulated the data collected above to the following six groups FGDs with HCWs from 18 CHCs and in-depth Interviews (IDIs) of three District Health Officers, five hospitals staff members, and a BPJS-Kesehatan representative at the next stage of this research.

The FGDs and IDIs were steered with open-ended questions and discussion guidelines. The guidelines consisted of questions to explore knowledge and awareness about TB-DM, existing TB-DM care and control strategies, problems and barriers impacting the delivery of services in private health care, and opportunities to develop comprehensive services for both diseases. The FGD guidelines were piloted prior to use with subjects with characteristics similar to those of the research participants.

Each FGD and interview took approximately 180 and 30–60 min, respectively. FGDs and IDIs used a combination of Indonesian and local Javanese languages. FGDs were held in meeting rooms in the District Health Office (DHO) area. IDIs were conducted in quiet room of the DHO, as chosen by the participants. The FGDs and IDIs were conducted separately at different times. All FGDs and IDIs were audio-recorded, and continued until saturation was reached, which indicated that no new theme emerged.

Data credibility was ensured during the FGD and interview process with multiple approaches. As described above, we conducted investigator triangulation, data source triangulation, and asked questions repeatedly by rephrasing them to confirm the participants’ answers [21]. All authors were involved in data collection. FGDs with the HCWs were conducted by DS and IP as moderators, while MA was an observer. The first author (MA) also performed IDIs with District Health Officers, hospitals staff members, and a BPJS-Kesehatan representative. All three authors have a background in qualitative research training and practices. MA and DS are female. MA is a medical doctor with an educational background in hospital administration, while DS has expertise in public health and nursing. IP is a male medical doctor and a researcher in the area of chronic care. We triangulated the data with data from the FGDs involving CHCs health workers and in-depth interviews with three regional health regulators, five hospitals staff members, and a BPJS-Kesehatan representative.

### Data analysis

The research assistant made careful verbatim transcriptions of the audio-recorded FGDs and IDIs. She has a health background and has also been trained in qualitative data collection. Transcripts were not given to the participants but were rechecked by MA and the research assistant before coding. Thematic analysis was applied using nVivo 12.0 to manage the coding, categories, and generation of the themes. The transcripts were read and reread to search for new trends and recurrent concepts. Codes were allocated to segments of the text, and similar codes were then assembled to form a category. Similar categories were then compiled to create a theme. The first author (MA) conducted the coding, while the others (DS and IP) reviewed the appropriateness of the coding and category and theme formulation. There was no significant dispute at the analysis stage, and all authors agreed to the themes developed.

## Results

Twenty-one HCWs from 13 clinics and eight GPs from solo practices attended the FGDs (Table 1). The age range of the participants was 26–69 years, with an average of 43.7 years. The majority of the participants were women (80.1%), with an average length of employment of 17.1 years (range = 2–25 years). The participants described current practices in the provision of TB-DM services and identified complex challenges affecting the integration of care for both disease care. They explored the opportunities for and potential roles of PPCs. The data analysis identified four main themes: health system-related barriers; HCW’s knowledge and perception; lack of implementation of bi-directional screening, and needs of multisector role.

**Table 1:** Socio-demographic characteristics of the participants

Participant’s code	Gender	Age (years)	Profession	Position	Length of employment (years)
***FGD of Clinic Representatives***					
R1	Female	30	GP	Health services manager	5
R2	Male	42	GP	Functional GP	2
R3	Female	30	GP	Functional GP	2
R4	Female	26	Midwife	Health service manager	4
R5	Female	45	GP	Functional GP	3
R6	Female	50	Midwife	Health services manager	20
R7	Female	55	GP	Head of clinic	4
R8	Female	32	GP	Functional GP	3
R9	Male	32	GP	Functional GP	2
R10	Female	37	Nurse	Health services manager	11
R11	Female	33	GP	Head of clinic	5
R12	Female	40	Nurse	Health services manager	21
R13	Female	54	GP	Head of clinic	25
***FGD of Solo General Practitioners***					
R1	Female	45	GP	Owner and functional GP	20
R2	Female	43	GP	Owner and functional GP	16
R3	Male	41	GP	Owner and functional GP	15
R4	Male	52	GP	Owner and functional GP	5
R5	Female	69	GP	Owner and functional GP	9
R6	Female	46	GP	Owner and functional GP	12
R7	Female	62	GP	Owner and functional GP	1
R8	Female	54	GP	Owner and functional GP	5

*GP* general practitioner

### Theme 1: health system-related barriers

In our study, private GPs/ solo providers more frequently found TB-DM cases among their patients than private clinics staff who have never had cases at all until these FGDs took place. Although all TB-DM cases were always referred to a hospital and/ or Puskesmas, some private-sector challenges related to TB-DM services were identified as health system-related barriers. These problems occurred due to the inadequate collaborative system in the transition from national into the local network. In this first theme, five categories were identified: the BPJS-Kesehatan procedures; health financing; diagnostic procedures; health facility networking; and human resources issues.

#### The BPJS-Kesehatan procedures

As the national social security agency for health, BPJS-Kesehatan has some mismatched regulations relating to the provision of the TB-DM services. Almost all participants in this study remarked that regulatory restrictions on non-specialist referrals made it challenging to refer patients for hospital examination/treatment. According to regulation, TB is a disease that must be managed by primary care facilities. The problem mainly occurs when DM patients have atypical TB symptoms and required a chest X-ray for TB screening. It is well-known that almost all primary healthcare facilities in Indonesia have no radio imaging equipment for these purposes. These barriers were also confirmed by HCWs from CHCs and hospitals.

*The problem will arise if the patient DM needs to be screened for TB. Not all of them have symptoms because there is an immune response, while the national consensus suggests using X-rays to check effusion or infiltrates. Meanwhile, the primary health facility did not have X-rays facilities, but referral (to the hospital) could not be done due to this BPJS regulation. (FGD of Clinics, R7)*

The complexity of electronic referral and re-referral forms that must be completed by doctors was also not acceptable. Some senior doctors often face barriers due to non-user-friendly applications or their lack of familiarity with new technology, i.e., in completing the TACC (Time-Age-Complication-Comorbidity) section for referring patients to hospital. Complicated procedures of patient referral sometimes led to the recording of incorrect diagnoses in referral forms, as disclosed by informants.

*There was a case yesterday. A patient was diagnosed with DM in my clinic, then he complained of prolonged cough, and so on. Then I referred him to the hospital with the same diagnosis that other doctors had reported earlier (Bronchitis). As we were not able to refer (to the hospital) with a diagnosis of TB, so we wrote Bronchitis." (FGD of Solo GPs, R6)*

In triangulation with the BPJS-Kesehatan representative, opportunities for discussion and piloting pathways for enhancing TB-DM case detection are still open. By applying national guidelines in TB-DM care and collaboration to mandatory chest X-rays for TB screening in DM patients, BPJS-Kesehatan representative identified potential barriers. These concerns regarding the increasing number of hospital patients and the risk of resources mobilized ineffectiveness compared to the screening results obtained. Hence, pilot testing of specific referral pathways for TB screening may be required to assess how these procedures affect health financing and case finding. On the other hand, BPJS-Kesehatan also recognizes the necessity of early detection of TB patients in primary care. Moreover, there are chances and needs to assess potential and risk for providing chest X-rays in primary care to resolve the gap.

#### Health financing

The private sectors practitioners mentioned interesting issues related to the potential burden of health costs beyond the national health insurance scheme. Since the TB programme was established in Indonesia, TB services have primarily been provided at CHCs as public health facilities to which private clinics and GPs can refer TB patients. Because the suspected TB patients’ participation in BPJS-Kesehatan is not registered at CHCs, specific procedures and services carry potential additional fees, i.e., registration/administration fees, and/or payment for supporting examination for DM or other health services. Presumption of health cost barriers arise because of patients’ inability or unwillingness to pay. In turn, these barriers prevent patients from accessing health services according to their current needs.

*The patient often say, “How much will it cost, Doc? If I am told to pay, I am still a (BPJS) member here ... .” So far, BPJS patients only know that BPJS participants should not pay anything. Anyway, they all know (that health services) should be completely free." (FGD of Solo GPs, R4)*

*Patients do not pay (for TB services at the Puskesmas), but every month a patient visits, they must register. Every time a patient registers, they have to pay, so instead of being complicated, it is advisable to move their BPJS membership to Puskesmas. (FGD of Solo GPs, R3)*

On the other hand, there are several types of PPCs located in Yogyakarta City. Differences in PPCs ownership, organisation/management, and membership coverage existed even before the universal health coverage (UHC) era. This situation has left a variety of health programmes and potential sources of health funding, in addition to the BPJS-Kesehatan scheme that could be used in TB-DM care and control.

*There are funds allocated to National Police officers for annual health checks, but this programme is not routine, either. It needs to be separated and sorted, and it takes turns for the individuals being examined. (FGD of Clinics, R11)*

*Because the company also covers our health costs, so even without BPJS, a patient can seek treatment outside. Yes, we discuss it there (with the patient). (FGD of Clinics, R9)*

#### Diagnostic procedures

The participants stated that chest X-ray screening, which is suggested by national guidelines, is procedurally constrained, as explained above. In general, private HCWs are not familiar with other TB tests, such as gen-Xpert/rapid molecular test, although DHO promoted gen-Xpert for increasing the detection of TB case in Yogyakarta. Instead of being more accessible, diagnosis by the sputum smear test was very challenging for DM patients. These constraints in diagnostic procedures were similar to the barriers identified in the triangulation with CHCs and hospitals staff members. The public sector faced the same barriers to accessing chest X-ray examinations and performing sputum sample collection for rapid molecular tests or sputum smears. Furthermore, PPCs participants conveyed that they always refer the patient to a third party if they require a supporting examination to validate a TB diagnosis. These conditions were challenging for proper and timely diagnosis in suspected patients.

*We are also in the same situation as other clinics that do not do supporting examinations by themselves. But, we cooperate with third parties, including for sputum examinations and X-rays. (FGD of Clinics, R9)*

#### Health facility networking

The participants highlighted the weakness in networking between health facilities. Although private health facilities cooperate in immunisation, diarrhoea management, and other national programs, collaboration for TB care and control are un-established. The majority of private HCW participants were not familiar with the concept of PPM; nor do they have a memorandum of understanding (MoU) for cooperation, mainly in TB management, with the local Puskesmas. However, discussions to initiate MoU formulation have taken place since accreditation of all primary health facilities was implemented.

Data recording and reporting are the other essential components that should be discussed in the development of an MoU between PPCs and Puskesmas. However, most participants expressed mixed feelings about the desire for an MoU to increase their authority in health services provision and potential obstacles that might arise. The strict reporting obligations, how to report, and the lack of reporting system between private and health facilities are complex challenges that need to be resolved.

*I do not know my obligation to report to the Puskesmas. This challenge is about how to establish cooperation with the Puskesmas. We are asked to propose (to the Puskesmas) MoU cooperation as required by BPJS and for TB eradication. Solo GPs also has to play a role, it must be a written MoU cooperation, and so far, I have tried to propose it, but the Puskesmas has not answered yet. The term formulation of MoU is still being discussed with the Health Office; what kind of cooperation is this? The formulation of cooperation is still in the process. (FGD of Solo GPs, R5)*

Another reported problem was communication and coordination between private and public primary care and hospitals. Although some of the GPs stated that they have an excellent relationship with public HCWs in general, they still experienced minimal feedback or responses from referral healthcare facilities. They never received feedback for some referrals, and they were lost during the follow-up of the patient.

*There is a TB patient who is also a BPJS participant we just handled once. And maybe we don't know the procedure because we just got it. If I am not mistaken for the treatment, this is provided in the Puskesmas near the patient's house; that is, the closest Puskesmas. Yesterday, because of the information from the Puskesmas near our clinic, the last case had to be reported. So, there was a miscommunication. That is our problem when there is no communication between the Puskesmas and our clinic. (FGD of Clinics, R2)*

The DHO and CHCs acknowledge that the private sector has not been involved in collaborative TB-DM care and control before the COVID-19 pandemic. From the perspectives of District Health Officers, the private sector did not seem to pay attention to government programmes. On the other hand, the DHO is preoccupied with many of the government’s health programme burdens, including achievement of target specified in the Minimum Service Standards. Hence, the DHO is still focused more on fostering Puskesmas/CHCs as Regional Technical Implementing Unit rather than the private sector.

*To be honest, because of the progress, yeah… TB-DM has not yet reached them* (PPCs)*. Because on the way, even on applying DOTS, their attention is low. So, we prioritize what we can do. (IDI of District Health Officer)*

#### Human resources issues

Although private HCWs showed positive attitudes by stating the possibility of assisting and being involved in TB-DM management, they still pointed out human resources issues, including the lack of staff assigned to TB programs. TB and TB-DM patients need to be adequately managed according to DOTS (Directly Observed Treatment Short-course) strategies. There was still a limited number PPC staff trained in DOTS/programmatic TB management. Meanwhile, some patients with chronic non-communicable disease were managed systematically with PROLANIS (BPJS chronic disease service programme), although it still needs improvement.

*Our problem is that there is no person in charge (PIC) for TB cases. For DM and hypertension patients that are members of PROLANIS, there is a designated appointed PIC. We do not have PIC for TB, but we have a quality and patient service department that will further explore TB cases even though we rarely have cases. DM and hypertension patient always managed and followed-up, but because TB patients go directly to the hospital, we don't have a TB PIC. (FGD of Clinics, R2)*

### Theme 2: HCW’s knowledge and perceptions

The participants showed wide variations in their baseline of knowledge and perspectives on TB-DM and its management. Three categories emerged; variation knowledge in TB-DM; false perceptions about TB-DM; and lack of TB information dissemination and training.

#### Variation of HCW’s knowledge about TB-DM

During the discussion, private HCWs showed diversity in their knowledge of the pathophysiology of TB-DM, treatment, types of screening, and standard treatment. Although most participants realised that DM was a risk factor for TB, none of them mentioned wether TB patients also have a higher risk of DM. Participants who expressed their opinions generally well understood the impact of DM on worsening outcomes for TB patients.

*Based on the theory, handling TB with DM or DM with TB will be more difficult. (FGD of Clinics, R10)*

*If I was asked about the relationship between DM and TB, in my opinion, they are related. Because it is associated with the immune system, which might at that time if we conduct anamnesis, it is indeed experiencing a decline in these patients." (FGD Clinics, R9)*

#### False perceptions about TB-DM

Health workers of some clinics perceived that TB-DM was rare because they had not or only seldom encountered a case. In the case of diagnostic procedure and treatment aspects, almost all private HCWs still maintained the impression that TB-DM is procedurally difficult to manage although various government policies allow this to be carried out. They believe that the patients cannot be given insulin if their HbA1c level is lower than 9%, according to the BPJS-Kesehatan requirement, although this requirement is not applicable to patients with comorbid conditions.

*As long as have I practised in the clinic, I have not found DM patients with TB symptoms. We haven't. I've never got a TB-DM case while practicing there. So, I haven't thought much about the relationship between the two diseases. (FGD of Clinics, R2)*

*This is another problem. Insulin can only be given if HBA1c is above 9. Yeah, that is just a new problem. (FGD of Solo GPs, R5)*

#### Lack of TB information dissemination and training

The variation in HCWs’ knowledge and perceptions may be due to a lack of training, information dissemination and coordination between PPCs, public sectors, and the DHO. Only one solo GP reported being invited to TB training in 2015. All private providers stated that they have never seen or read the guidelines or national consensus on TB-DM. This situation also led to HCWs being unaware of the importance of bi-directional screening, regimen standards, and integrative care for TB/ TB-DM patients.

*The Health Office is already undertaking a lot of accreditation-related information dissemination intensively. But, in my opinion, information regarding new guidelines and regulations (about TB-DM) is still limited. (FGD of Clinics, R2)*

### Theme 3: the lack of bi-directional screening implementation

The participants stated that TB-DM screening has not yet begun to be applied systematically in daily practice. This situation is described under three categories in this theme: screening pathway; screening difficulties; and screen opportunities.

#### Screening pathway

None of the private HCWs stated that the bi-directional screening was being done comprehensively and regularly carried out in their health facilities. TB screening in DM patients was performed sporadically, and patients were only superficially investigated if they complained symptoms. This screening was usually performed based on the physician’s clinical judgment or if the symptoms suggested suspected TB.

*… the problem is (only) if there are complaints, we then anticipate it. Complaints of cough are rarely or not become the patient's main reason to come for treatment. There could be inaccuracies or inadequacies during history taking due to many patients, long queues, and many other reasons. So, we only explore the primary diagnosis (DM) or if the patient does not appear to be coughing in the room. It is considered sufficient only to ask about cough complaints, especially in DM patients who are generally only checked for blood glucose then asked for the referral form and finished. It's not well organised. (FGD of Clinics, R6)*

#### Screening difficulties

Informants noted that some factors could become obstacles in implementing bi-directional screening. It was highlighted that DM patients often have atypical symptoms or cannot expectorate or produce sputum for smear examination/rapid molecular test. The difficulty of accessing X-rays also remains a significant obstacle in implementing bi-directional screening. On the other hand, patients who refuse to be referred for diagnostic procedures might also delay the diagnostic examination. Moreover, HCWs of CHCs also confirmed similar barriers to screening.

*It's not easy to diagnose TB, either because there are many elderly patients who say that, “When I'm old, it's usual to have a prolonged cough.” There are still a lot of opinions like that. “It's a cough because I'm an old man.” So he came back two months or three months later. The cough is not cured, or the child is sick, the family members are sick, the grandchild is sick. It turned out that his grandparent was ill first." (FGD of Clinics, R3)*

*The X-ray might facilitate the technique, whereas the sputum screening test is difficult. (FGD Private GPs, R3)*

#### Screening opportunities

Despite the complicated existing obstacles, the participants identified several opportunities to screen for TB in DM patients. Some routinely performed activities can be used for intensive TB screening, i.e., initial assessments by HCWs in health facilities, general routine check-ups, and Posbindu/ Posyandu (integrated community health services post).

*We also carry out routine activities for the National Police, which are periodic for POLRI (The State Police of Republic of Indonesia) members themselves, as well as X-rays and so on. (FGD of Clinics, R11)*

Some participants also reported that ‘fee for service’ or non-BPJS patients were more flexible about taking up the screening procedure. Some patients would usually pay for diagnostic procedures suggested by the doctor.

*Our advice to patients is, for example, “If you haven't had an X-ray for the last three months, I recommend X-rays.” If he is not a BPJS patient, it will be more comfortable, but for this BPJS patient, it's still a bit difficult to do. (FGD of Clinics, R7)*

### Theme 4: the needs of multisector roles

TB-DM patients need to be managed collaboratively. Hence, the functions of stakeholders, health facilities, and health organisations/associations have a crucial part to play. Many DM patients seek treatment in private health facilities. Meanwhile, to involve and engage private sector, the need for multisector support was identified.

This theme is organised into five categories: roles of the DHO, roles of health facilities/health profession associations; patients’ roles; potential roles of PPCs; and encouragement by BPJS-Kesehatan.

#### Roles of the district health office (DHO)

The DHO has key roles in encouraging the private sector to be involved in TB-DM collaboration. At the time of these FGDs, TB-DM information dissemination to PPCs had not yet started; hence the DHO should ensure that all health facilities that serve patients with TB/DM are well informed about this new programme. Such a strategy could use the DHO, e.g., by engaging healthcare/professional associations.

*If I may suggest how if the Health Office cooperates with professional organisations in information dissemination regarding new guidelines, new regulation, or the programme of the DHO itself in the city. Because ASKLIN (private clinics associations) already accommodates almost all private clinics in their respective cities. So, in my opinion, if the DHO reaches them, it will be much easier than the DHO approaching private primary care one by one. (FGD of Clinics, R2)*

In addition to information dissemination and connecting or coordinating with relevant organisations, systematic monitoring of health facilities should be undertaken to ensure the programme implementation. Greater effort at direction, monitoring, and evaluation of the private sector is required to implement the programme. PPCs also need to advocate about an appropriate payment mechanism.

*We have come to attend DHO information dissemination on other topics, but usually just delivering information. We do not get any guidance or monitoring and so on to ensure its implementation. (FGD of Solo GPs, R9)*

*We have not done that screening. Because clinics are also not burdened for screening, right? Well, who pays the fee? So, if there are clinical symptoms that support the suspicion of the disease, we refer (to hospital/ Puskesmas). (FGD of Clinics, R2)*

#### Roles of health facilities or health professional associations

Asosiasi Klinik Indonesia (ASKLIN), or association of private clinics, which represents all private clinics in Yogyakarta City, has not been involved in initiating the collaborative TB-DM programme in private health facilities. This organisation also focuses more on advocacy or assistance for members on accreditation and issues related to BPJS-Kesehatan rather than support from other government programmes.

*Until now, the focus of ASKLIN is still on accreditation related to the BPJS requirements that in 2021 all health facilities must be accredited. …. As far as I know, ASKLIN, the focus is still more on accreditation. As for seminars or programs, there is not yet a particular program for it (TB-DM)." (FGD of Clinics, R6)*

ASKLIN or other similar organisations could play a role in increasing private sector engagement with government programmes. Until now, there has been reasonably good collaboration between the public and private sectors in a limited number of programmes, such as immunisation and DHF (dengue hemorrhagic fever). Although the TB programme has been initiated in various Public-Private Mix (PPM) activities, its implementation is not progressing well. Hence, ASKLIN can play a role in designing the MoU of cooperation between PPCs and government health facilities and conducting various educational sessions or publicising national programmes to its members.

*ASKLIN's role was included due to the BPJS credentialing this year which requires an MOU with the Puskesmas so that ASKLIN then intervened in the clinic's relationship with the Puskesmas* (CHC) *because before that it was only independent between the clinic and the Puskesmas concerned. (FGD of Clinics, R2)*

The role of health professional organisations similar to ASKLIN could be an extension of the role of the DHO to disseminate information about priority programmes. IDI/ Ikatan Dokter Indonesia (Indonesian Medical Association), specialist doctors’ associations, and other HCW organisations have not been much involved until now. However, health professional organisations have often been invited to several national-level discussions to reach a consensus.

*… In my opinion, it is much easier than the DHO calling or coming to each private primary health facility. Because it does not represent all primary care, maybe if the health office calls the health facilities, the DHO will invite large health facilities or from a certain area only. But if DHO can hold talks with ASKLIN or professional organisations, all of them would be accommodated. It is more coordinated. (FGD of Clinics, R2)*

#### Patients’ roles

Patients also have essential roles in the successful collaborative TB-DM care and control. Patients’ key roles include complying with treatment and following doctor’s advice, involvement in decision-making, and willingness to pay some healthcare costs and other financial burdens related to diagnosis and treatment not covered by the insurance scheme.

*Yes, we first check the X-Ray. In our clinic, when there are X-ray and sputum data that show TB, we will discuss it with the patient. … because of the absence of drugs and so on, we will discuss whether he is willing to be referred (to other health facilities) because the BPJS is not used here. (FGD of Clinics, R9)*

#### Potential roles of the private sectors

Participants mentioned some activities that are routinely conducted by PPCs. Some activities were listed as potential roles that they can take on if involved in collaborative TB-DM control and care. Private health facilities commonly have a large number of DM patients. Hence, health promotion and patient education become a significant factor for prevention, increasing patient compliance with treatment, and patient referral. The versatility of BPJS membership and characteristics of the partnership between patients and PPCs are also opportunities for programme success. In general, patients have a close relationship with health workers, and PPCs are also highly accessible. National insurance membership is also quite flexible, which means that participants can temporarily move their health facility membership during TB treatment to Puskesmas or other DOTS facilities if they are worried about additional health service costs.

*We are closer to the patient so that they are better able to warn against routine treatment because usually… those with chronic comorbid diseases routinely come every month. We can request from them that even if they take medicine at the hospital with a control letter, “If anything happened, please come to our practice!” If they want a consultation, please come, and they are happy. They sometimes meet a specialist doctor in the hospital, but he doesn't even touchthem because there are so many patients. And by that way, we can monitor them. (FGD of Solo GPs, R4)*

According to the participants, referral to DOTS facilities is the only action that can currently be taken by PPCs after identifying suspected TB-DM patients. This situation has beenc caused by several issues related to PPCs’ authority, including untrained staff, unavailability of TB drug regimens according to the current ISTC standards, and the inability to prescribe insulin. When patients are finally referred to other health facilities, PPCs often have difficulty in following up the patients’ treatment because of minimal feedback and coordination, as stated above. Thus, challenges on continuity of care have also emerged as obstacles.

*We were unable to order TB drugs. We detected the TB patients who came to us with cough through anamnesis. Then, even for sputum smear examination, we also referred to a third party. After he is positive for TB, we will return him or educate him to take medication at the Puskesmas in the local area where the patient lives. However, we are not very involved in monitoring TB patients. (FGD of Clinics, R2)*

Although some barriers are mentioned above, private providers expressed their mixed feelings about their ability to be involved in successful TB-DM collaboration. Due to being customer-satisfaction-oriented, they intend always to pay attention to every clinical complaint with appropriate follow-up. However, they also indicated their reluctance to conduct home visits, which are usually required in TB management, due to the high workloads.

*… if I am asked whether or not this programme can run actively in private health facilities, I can say that it can indeed run actively. Because we, from the private sector, always try not to ignore patient complaints. It will be followed up unless there are no complaints. (FGD of Clinics, R6)*

*Even if we are asked for assistance to make a report as long as we can do it, I think the private sector can be quite helpful, as long as we are not asked to visit the patient at home. It will be a hassle. No problem… because I think the private sector will not mind if the Puskesmas can more actively involve us. (FGD of Clinics, R2)*

#### Encouragement by BPJS-Kesehatan

Since BPJS-Kesehatan’s role is as a significant payor in the national health system, it has encouraged the well running of several national programmes. Comprehensive care through PROLANIS (chronic care program for DM and hypertension) is one of the commitment-based PPC performance assessment criteria. Participants pointed out their expectations regarding BPJS-Kesehatan’s potential role in encouraging regular screening for TB in DM patients.

*BPJS should have regulations to ensure that there must be TB screening in DM cases, for example, in the PROLANIS program. Every six months, there is always an HbA1C examination, complete lipid profile, and urea creatinine. Thus, TB screening could be included in this six-month programme, for example. (FGD of Clinics, R7)*

## Discussion

This study is the first to explore from the PPCs’ perspective existing practices and readiness of Indonesia to implement collaborative TB-DM care and control. In general, our study showed that PPCs have not started to be involved in collaborative TB-DM management. PPCs lack the opportunities to play active roles in collaborative TB-DM management due to many limitations. However, PPCs have the potential to conduct health promotion for TB-DM, bi-directional screening, treatment, referral, and reporting within an adequate capacity-building programme and logistic supplies.

This study indicates that TB-DM partnership could be more robust by PPM scaling up and increasing assistance and supervision of PPCs. The DHO’s role in regional health management becomes crucial in establishing collaborative relationships between various sectors. However, the collaborative system has not been developed yet, creating obstacles to integrating programmes, including PPM [22, 23]. As indicated in the literature, the effectiveness of achieving the desired global targets in TB control is contingent on an effective network of high-quality and comprehensive health services [19, 24]. Hence, stakeholder agreements and promotion of their ownership are necessary to support collaboration [22, 25]. Strategies differentiated by region may also be needed to respond to the variation in trends in patients’ care-seeking and enhance private and public providers’ capacity [16, 23, 26, 27]. Thus, raising HCWs’ awareness and improving their quality of care are essential rather than just getting them to disseminate information [27].

Our study showed that providing care for TB-DM patients often requires health facilities and professionals to collaborate. However, some health services are not accessible due to some health financing restrictions and standard operating procedures. Our study also indicates the need for further involvement of BPJS-Kesehatan in spurring the implementation of collaboration. It is important to note that the impacts of BPJS-Kesehatan/national health insurance regulation on care pathways greatly influence healthcare providers’ ability to meet the established indicators. On the other hand, although the National TB Program (NTP) covers microscopic examination and TB drug regimen funding, the particular condition of atypical TB symptoms in DM patients needs other procedures, such as chest X-ray, that are not covered by insurance or the programme [12, 18]. Hence, the formulation of a national TB-DM collaborative system that involves major payor agencies and private health service providers is strongly recommended to address the challenges, especially since several studies have shown that the sub-optimal outcomes of PPM are due to lack of funding and weak governance [18, 23, 28].

Another important finding was that patients have vital roles in the success of TB-DM care and control. Consistent with previous findings, this study revealed the need to improve patient knowledge as a prerequisite to ensure their compliance with health care requirements [29, 30]. Hence, self-management support and patient-centered care (PCC) may support the successful TB-DM care services as it becomes the critical component in the multimorbid disease care frameworks [9, 31, 32]. On the other hand, our study also revealed that patients’ potential cost burdens to access health services also have an impact on timely diagnosis and subsequent appropriate treatment [9]. Thus, patients’ complex bio-psycho-socio-economic problems should be solved to strengthen the medical treatment approach [24, 33]. This finding has important implications for developing a strong connection between private sectors providers, government agencies, and community support providers, i.e., community health workers, and non-governmental organisations (NGOs), etc. [19].

This study shows that operational constraints in collaborative TB-DM care and control are more prominent in TB case finding and management. A possible explanation of these problems is the existence of various interacting factors that affect TB-DM care, such as the complexity of the patient’s condition, the lack of capacity of PPCs’ HCWs in TB management, PPCs lack of implementation of the TB programme, and the complexity of service procedures. The identified barriers to TB screening in PPCs are in line with the findings of relevant studies in other countries. In Pakistan, the existing obstacles to bi-directional screening include difficulties in diagnosing TB in DM patients with atypical TB symptoms, patients’ inability to expectorate sputum, the cost of chest X-ray, and the distance to the referral health facilities for diagnostic evaluation [34]. In India, challenges of the TB-DM screening implementation include additional fees, additional visits for diagnostic purposes, poor feedback from referral health facilities, the workload of HCWs, and the need for additional records and reporting [22].

One of the crucial findings of this study is that private providers may doubt the effectiveness of bi-directional screening due to their perceptions of low incidence of TB in DM patients seen by them. Instead, there is debate on how to conduct efficient and effective bi-directional screening [27]. Considering that health workers are the most important stakeholders for the effective delivery of integrated health services, various efforts are needed to prevent their resistance [35].

These study results also agree with the findings of recent studies showing that health professionals prepared to manage TB have not been adequately supported in the private health sector [14, 16, 26]. Insufficient capacity due to private providers without training in TB management is a common problem in many countries that have initiated collaborative TB-DM management. Lack of training in TB screening procedures is also reported in India [22]. In Ethiopia, HCWs indicated a lack of knowledge and skills in DM management [35]. Nigeria also reported a lack of providers’ knowledge of diagnostic and non-pharmacological treatment for DM [36]. Filling the gaps in private providers’ perception, knowledge, and skill is crucial to the appropriate set-up of collaborative TB-DM care and control [37].

The high risk of disruption of the continuity of TB-DM care in the private sector is also a challenge. As shown by previous TB patient pathway research in Indonesia, PPCs in our study do not hold supporting examinations for TB by themselves due to the unavailability of laboratory and X-ray equipment [16]. They also do not have supplies of standard drugs for TB treatment based on ISTC (International Standard for Tuberculosis Care) 2019. In addition, insulin for DM treatment can only be prescribed by an specialist in referral hospitals or drug stores collaborating with the BPJS-Kesehatan’s Back-Referral Program. These incomplete and fragmented services at PPCs lead to low quality and delayed delivery of TB-DM care.

While collaborative TB-DM care and control has not yet been adequately developed in PPCs, our study shows that DM is beginning to be comprehensively managed in Indonesia. Since BPJS-Kesehatan launched PROLANIS (chronic disease care program), PPCs have developed better-organised DM services. In contrast, report on Nigeria suggest unpreparedness and inappropriate DM management as the major problems in initiating collaborative TB-DM management; the unavailability of a continuity-of-care system for DM, providers’ inadequate knowledge of and skills for managing DM, recurrent stockouts of DM supplies, the inability of patient to pay for DM services, ineffective DM data management, and lack of government prioritisation of DM care have been noted [37]. A study of TB management in India reported the same issues and highlighted the lack of standardised DM treatment as a crucial challenge in collaborative TB-DM management [22]. DM is generally not managed programmatically like TB. Hence, one of the most important aims of the WHO TB-DM Collaboration for Care and Control initiative is to enhance DM management by co-learning with TB management [5, 27].

It is interesting to note that the opportunities to involve the private sectors in collaborative TB-DM care and control are widely available. Flexibility in various healthcare funding sources, the adjustable care pathway of non-BPJS patients, pre-existing routine health screening activities, a close relationship between private providers and patients, and the ability to transfer BPJS membership are opportunities to overcome barriers. Despite their limitations, PPCs, as gatekeepers of Indonesia’s health system could implement collaborative TB-DM management as part of their main health prevention and promotion function. PPCs could perform TB treatment for DM patients, conduct high-quality referrals, and provide data reporting TB-DM programme indicators within adequate capacity-building programmes and logistic supplies.

The findings of this study indicate the importance of taking a multi-faceted approach to initiate collaborative TB–DM management in PPCs. The lessons learned from the successful integration of the TB–HIV programme are the need for solid instructions from the government [38] and several adjustments to applicable national standards [28]. In terms of screening, for example, sub-Saharan African countries include a TB symptom questionnaire as a routine screening activity for people with HIV [28]. This method is feasible and finds many new TB cases. In addition, HCWs accepted the TB screening process as part of the existing HIV documentation/reporting process. Thus, simplification of collaborative procedures and prevention of duplication of activities/reporting should increase programme acceptance and implementation.

One of the issues that emerged from this study is the necessity for PPCs to access supporting examination facilities for TB diagnosis and high-quality treatment. Lessons learned from the factors leading to the successful integration of the TB-HIV programme were also related to the availability of screening facilities for TB, including increased access to chest X-rays [39]. These efforts have succeeded in improving the diagnosis of TB cases, although not of patients with active cough symptoms. In addition, with regard to the quality standards of healthcare services, maintenance of the follow-up of HIV patients after completing TB treatment and good documentation and reporting for evaluation and decision-making related to policies and financial support also enabled the successful integration of the two programmes [39].

This study has several strengths. The qualitative method adopted by the study enabled in-depth exploration of providers’ perspectives and provided a rigorously collected set of rich data owning to efforts to maintain trustworthiness. Although this study has clearly defined the current private sector situation in collaborative TB-DM care and control provision, several limitations should be considered. The data interpretation is contextualised; therefore, relevant quotations were used to describe their applicability to similar health system settings. Additional data from observations on care delivery in PPCs, document review, and other quantitative inquiries may be needed to more comprehensively assess service provision and programme management of both diseases.

## Conclusions

This study provides insights into emerging health system barriers and opportunities that need to be addressed in integrating TB-DM services. There are possible obstacles due to a lack of infrastructure, human resources, and healthcare coordination. The results indicate a wide range of opportunities to incorporate the TB control program into current DM treatment in PPCs. Due to the limitations of PPCs, their potential roles in carrying out health promotion, bi-directional screening, patient referrals, TB treatment, and data reporting should be continuously improved.

Therefore, it is essential to increase PPC involvement by enhancing their capacity and implementing PPM. PPC engagement should be initiated and maintained with multisector support to ensure the sustainability of the programme Further work is required to pilot integrated TB and DM services in private healthcare facilities according to their potential roles. Assessment of the feasibility for possible collaboration between services for treatment of the two comorbidities is needed to integrate the two programmes.

## Data Availability

The datasets used and/or analyzed during the current study are available from the corresponding author on reasonable request.

## Abbreviations

ASKLIN: Asosiasi Klinik Indonesia (Indonesian Clinics Association)
BPJS-Kesehatan: Badan Penyelenggara Jaminan Sosial-Kesehatan (national health insurance)
CHC: Community Health Center
DHO: District Health Office
DM: Diabetes mellitus
DOTS: Directly Observed Treatment Short-course
FGD: Focus group discussion
HIV: Human Immunodeficiency Virus
HCW: Health care worker
IDI: In-depth interview
ISTC: International Standard for Tuberculosis Care
GP: General practitioner
MCH: Maternal-child health
NGO: Non-govermental organisation
MoU: Memorandum of understanding
NHI: National Health Insurance
NTP: National TB programme
PIC: Person in charge
Posyandu: Pos Pelayanan Terpadu (integrated service post)
PPC: Private primary care
PPM: Public-Private Mix
TACC: Time-Age-Complication-Comorbidity
TB: Tuberculosis

## References

[1] International Union Against Tuberculosis and Lung Disease and World Diabetes Foundation, “Bali Declaration on the Looming TB-Diabetes Co-Epidemic,” IUALTD and WDF. 2015. pp. 1–3. [Online]. Available: http://www.theunion.org/bali-declaration.pdf.

[2] WHO (2018). The top 10 causes of death.

[3] WHO. Global Tuberculosis Report 2020. Geneva; 2020. [Online]. Available: https://www.who.int/publications/i/item/9789240013131

[4] Workneh MH, Bjune GA, Yimer SA (2017). Prevalence and associated factors of tuberculosis and diabetes mellitus comorbidity: a systematic review. PLoS One.

[5] WHO (2011). Collaborative framework for care and control of Tuberculosis and Diabetes. World Health.

[6] Lönnroth K, Roglic G, Harries AD (2014). Improving tuberculosis prevention and care through addressing the global diabetes epidemic : from evidence to policy and practice. Lancet Diabetes Endocrino.

[7] Alisjahbana B (2006). Diabetes mellitus is strongly associated with tuberculosis in Indonesia. Int J Tuberc Lung Dis.

[8] Yorke E, Atiase Y, Akpalu J, Sarfo-kantanka O, Boima V, Dey ID (2017). The bidirectional relationship between tuberculosis and diabetes. Tuberc Res Treat.

[9] Arini M, Ahmad RA, Utarini A (2020). Tuberculosis and type 2 diabetes mellitus (TB-DM) comorbidity care: barriers from the patients’ perspective. Enferm Clin.

[10] International Diabetes Federation. IDF Diabetes Atlas Ninth edition 2019. 2019. pp. 1–176. [Online]. Available: https://diabetesatlas.org/idfawp/resource-files/2019/07/IDF_diabetes_atlas_ninth_edition_en.pdf.

[11] BPJS Kesehatan. Panduan Praktis PROLANIS (Program Pengelolaan Penyakit Kronis). Jakarta: BPJS Kesehatan; 2015. pp. 1–10. [Online]. Available: https://bpjs-kesehatan.go.id/bpjs/dmdocuments/06-PROLANIS.pdf.

[12] Trisnantoro L, Herbianto D, Asrullah M, Sulistiawan D, Ekadinata N. Analisis Kebijakan Pembiayaan Program TB Dalam 3 Tahun Pelaksanaan Jaminan Kesehatan Nasional. Yogyakarta: PKMK FK-KMK UGM; 2017. pp. 1–32. [Online]. Available: https://kebijakankesehatanindonesia.net/datakesehatan/file/Laporan-reviu-kebijakan-program-JKN.pdf.

[13] Mahendradhata Y (2017). The Republic of Indonesia Health System Review. Heal Syst Transit.

[14] Mahendradhata Y (2015). How do private general practitioners manage tuberculosis cases? A survey in eight cities in Indonesia public health. BMC Res Notes.

[15] Ahmad RA, Mahendradhata Y, Utarini A, de Vlas SJ (2011). Diagnostic delay amongst tuberculosis patients in Jogjakarta Province, Indonesia is related to the quality of services in DOTS facilities. Trop Med Int Heal.

[16] Surya A (2017). Quality tuberculosis Care in Indonesia : using patient pathway analysis to optimize public – private collaboration. JID.

[17] Mahendradhata Y, Utarini A, Lazuardi U, Boelaert M, Stuyft PVD (2007). Private practitioners and tuberculosis case detection in Jogjakarta, Indonesia: actual role and potential. Trop Med Int Heal.

[18] USAID (2018). Engaging Private Providers to Improve TB Outcomes in Indonesia.

[19] WHO. Public-private mix for TB care and control: a toolkit. Geneva: WHO; 2010. p. 1–66. [Online]. Available: https://apps.who.int/iris/bitstream/handle/10665/44450/9789241500487_eng.pdf?sequence=1&isAllowed=y.

[20] Dinas Kesehatan Kota Yogyakarta. Profil Kesehatan Kota Yogyakarta Tahun 2019 (Data Tahun 2018). Yogyakarta; Dinas Kesehatan Kota Yogyakarta; 2019. pp. 1–262. [Online]. Avalibale: https://kesehatan.jogjakota.go.id/uploads/dokumen/profil_dinkes_2019_data_2018.pdf.

[21] Carter N, Bryant-lukosius D, Dicenso A, Blythe J (2014). The use of triangulation in qualitative research. Methods Meanings.

[22] India Tuberculosis-Diabetes Study Group (2013). Screening of patients with tuberculosis for diabetes mellitus in India. Trop Med Int Heal.

[23] Lei X, Liu Q, Escobar E, Philogene J, Zhu H, Wang Y (2015). Public–private mix for tuberculosis care and control : a systematic review. Int J Infect Dis.

[24] Mahendradhata Y, Lambert M-L, Van Deun A, Matthys F, Boelaert M, van der Stuyft P (2003). Strong general health care systems: a prerequisite to reach global tuberculosis control targets. Int J Health Plann Manage.

[25] Naqvi SA (2012). Implementing a public-private mix model for tuberculosis treatment in urban Pakistan: lessons and experiences. Int J Tuberc Lung Dis.

[26] Gede IW (2013). Factors associated to referral of tuberculosis suspects by private practitioners to community health centres in Bali Province , Indonesia. BMC Health Serv Res.

[27] Harries AD (2015). Diabetes mellitus and tuberculosis: programmatic management issues. Int J Tuberc Lung Dis.

[28] Howard AA, El-Sadr WM (2010). Integration of tuberculosis and HIV services in sub-Saharan Africa: lessons learned. Clin Infect Dis.

[29] Agrimon OH. Exploring the feasibility of implementing self-management and patient empowerment through a structured diabetes education Programme in Yogyakarta City Indonesia: a pilot cluster randomised controlled trial. Adelaide: The University of Adelaide; 2014. pp. 1–25. [Online]. https://digital.library.adelaide.edu.au/dspace/bitstream/2440/87696/8/02whole.pdf.

[30] Ting X, Yong B, Yin L, Mi T (2016). Patient education and counseling patient perception and the barriers to practicing patient-centered communication : a survey and in-depth interview of Chinese patients and physicians. Patient Educ Couns.

[31] Poitras M, Maltais M, Bestard-denommé L, Stewart M, Fortin M (2018). What are the effective elements in patient-centered and multimorbidity care ? A scoping review. BMC Health Serv Res.

[32] Barr VJ (2003). The expanded chronic care model: an integration of concepts and strategies from population health promotion and the chronic care model. Hosp Q.

[33] Boehmer KR, Dabrh AMA, Gionfriddo MR, Erwin P, Montori VM (2018). Does the chronic care model meet the emerging needs of people living with multimorbidity? A systematic review and thematic synthesis. PLoS One.

[34] Basir MS, et al. Operationalization of bi-directional screening for tuberculosis and diabetes in private sector healthcare clinics in Karachi, Pakistan. BMC Health Serv Res. 2019;19(1). 10.1186/s12913-019-3975-7. 10.1186/s12913-019-3975-7 PMC6404337 30841929

[35] Workneh MH, Bjune GA, Yimer SA (2016). Assessment of health system challenges and opportunities for possible integration of diabetes mellitus and tuberculosis services in south-eastern Amhara region, Ethiopia: a qualitative study. BMC Health Serv Res.

[36] Ogbera OA, Adeyeye O, Odeniyi IA, Adeleye O (2013). Knowledge of diabetes mellitus in tuberculosis amongst healthcare workers in Nigeria. Indian J Endocrinol Metab.

[37] Li L (2012). Screening of patients with tuberculosis for diabetes mellitus in China. Trop Med Int Heal.

[38] Nicholson T, et al. Report and Analysis Toward comprehensive global health care delivery: Addressing the double threat of tuberculosis and diabetes. 2017. [Online]. Available: http://www.advanceaccessanddelivery.org/news/2017/10/5/aad-report-highlights-pioneering-efforts-imagines-path-toward-comprehensive-global-care-delivery-for-tuberculosis-and-diabetes.

[39] The Union. Implementing Collaborative TB-HIV Activities: A Programmatic Guide. 2012. [Online]. Available https://www.theunion.org/what-we-do/publications/technical/english/pub_tb-hivguide_eng_web-1.pdf.

